# Effects of Short Term Bioturbation by Common Voles on Biogeochemical Soil Variables

**DOI:** 10.1371/journal.pone.0126011

**Published:** 2015-05-08

**Authors:** Burkhard Wilske, Jana A. Eccard, Marcus Zistl-Schlingmann, Maximilian Hohmann, Annabel Methler, Antje Herde, Thilo Liesenjohann, Michael Dannenmann, Klaus Butterbach-Bahl, Lutz Breuer

**Affiliations:** 1 Department of Landscape, Water and Biogeochemical Cycles, Institute for Landscape Ecology and Resources Management (ILR), Research Centre for Bio Systems, Land Use and Nutrition (IFZ), Justus Liebig University, Giessen, Germany; 2 Institute for Animal Ecology, University of Potsdam, Potsdam, Germany; 3 Karlsruhe Institute of Technology KIT/IMK-IFU, Garmisch-Partenkirchen, Germany; 4 International Livestock Research Institute, Nairobi, Kenya; University of Copenhagen, DENMARK

## Abstract

Bioturbation contributes to soil formation and ecosystem functioning. With respect to the active transport of matter by voles, bioturbation may be considered as a very dynamic process among those shaping soil formation and biogeochemistry. The present study aimed at characterizing and quantifying the effects of bioturbation by voles on soil water relations and carbon and nitrogen stocks. Bioturbation effects were examined based on a field set up in a luvic arenosol comprising of eight 50 × 50 m enclosures with greatly different numbers of common vole (*Microtus arvalis* L., ca. 35–150 individuals ha^–1^ mth^–1^). Eleven key soil variables were analyzed: bulk density, infiltration rate, saturated hydraulic conductivity, water holding capacity, contents of soil organic carbon (SOC) and total nitrogen (N), CO2 emission potential, C/N ratio, the stable isotopic signatures of ^13^C and ^15^N, and pH. The highest vole densities were hypothesized to cause significant changes in some variables within 21 months. Results showed that land history had still a major influence, as eight key variables displayed an additional or sole influence of topography. However, the δ^15^N at depths of 10–20 and 20–30 cm decreased and increased with increasing vole numbers, respectively. Also the CO2 emission potential from soil collected at a depth of 15–30 cm decreased and the C/N ratio at 5–10 cm depth narrowed with increasing vole numbers. These variables indicated the first influence of voles on the respective mineralization processes in some soil layers. Tendencies of vole activity homogenizing SOC and N contents across layers were not significant. The results of the other seven key variables did not confirm significant effects of voles. Thus overall, we found mainly a first response of variables that are indicative for changes in biogeochemical dynamics but not yet of those representing changes in pools.

## Introduction

Bioturbation is the directed disturbance of the pedosphere by biota. It can alter fundamental soil properties such as porosity, particle-size distribution, creep flux rate, and nutrient contents [1]. With respect to the active translocation of matter by soil-dwelling rodents (3.4–57.4 m^3^ ha^–1^ yr^–1^, [2]), bioturbation appears as one of the most dynamic processes among the mostly slow drivers that shape soil characteristics and biogeochemistry.

Although bioturbation is recognized an almost ubiquitous process contributing to important ecosystem services [3–6], the bulk of present day literature about terrestrial bioturbation is still mainly focused on invertebrate activity. This emphasis on the smaller rather than the larger animals involved in bioturbation may be partly the aftermath of man’s agricultural history, in which small rodents were only perceived as a threat [7]. However, the few studies with burrowing mammals clearly demonstrate that they dominate bioturbation and have a high impact on soil mixing and soil formation under polar, montane, semi-arid and arid climate conditions, i.e., in >60% of all terrestrial ecozones [1, 8–10]. In many places, Microtine rodents, i.e. voles and hamsters, seem to contribute the major share of vertebrate bioturbation [1]. This applies especially to the large steppe ecosystems as well as climate and management sensitive mountain meadows [11–13].

Today, global change requires reassessment of resources, and various studies have already shed light on the involvement of soil-dwelling rodents in ecosystem functioning and related biogeochemical cycles [14]. Smaller soil-dwelling vertebrates including rodents were reconsidered for significantly affecting plant biomass [15], plant community composition [15–17] and dynamics [18, 19], sediment movement [20], soil aggregates and nutrient development [21–23] and water relations [24].

Changes in some of the soil variables, which are affected by burrowing rodents, can interfere directly with efforts of agricultural and societal adaption to climate change. Above all, burrowing rodents may shift biogeochemical fluxes and pools of water, carbon (C), and nitrogen (N), and thereby affect (a) the drought sensitivity of soils, and (b) their sink and source functions for carbon and the three major greenhouse gases CO_2_, CH_4_, and N_2_O. Thus, the controversy between pest control and conservation of biodiversity unfolds on a new level, and the effects of burrowing rodents on the cycles of C, N, and water need urgently be quantified.

Pastor et al. [25] suggested that voles contribute more to nutrient fluxes than larger herbivores, because of the large vole populations, and because their small-grained and evenly spread feces are processed faster. The residence times of vole fecal N ranged only in the order of a few days [25], and in consequence, vole activity increased soil mineral N content. Furthermore, concentrations of soil nitrate were found to be elevated in soils that are populated by larger animal densities [21, 22, 26, 27]. This indicates that feces released by small mammals are affecting soil microbial processes, mineralization and nitrification, and thus inorganic N accumulation and the distribution/dispersion of nutrients across the soil. Small mammals add to local soil fertility not only through their excretion, but also due to the construction of foraging pits, which function as resource traps for sediments (subterranean erosion), litter, seeds and nutrients [20, 28, 29]. The laboratory and model study by Clark et al. [22] suggested that the minimum fecal and urine N-contribution of a free-ranging mixed population of small murid rodents was equivalent to 74% of N_2_ fixation, 7–9% of plant N uptake, and 19–37% of the atmospheric N deposition at reference plots of the Konza Prairie, Kansas. Furthermore, Bakker et al. [21] found a 1.5-fold increase in net annual N mineralization in N-limited grassland plots exclusively populated by common vole as compared to a surrounding area where voles, rabbits and cattle grazed jointly. The increase was mainly due to an autumn peak in N-mineralization, in which however both, vole activity and biomass input not grazed by cattle may have had their influence.

Obviously, experiments at various scales are required to fully comprehend the effects of voles in the complex setting of interacting pools and processes including feedbacks [1]. To our knowledge, field enclosures adequate to vole populations have not yet been employed to study the vole effects on soil biogeochemistry. The aim of the present study was to fill the gap between laboratory cages with controlled in- and output (e.g. [22, 30]) and rodent-permeable exclosure experiments with unrestricted fluxes [16, 17, 21, 31]. The objective was to obtain a snapshot of both the dynamics and the pools in order to complement the understanding that can be derived from other scale and/or model approaches.

We used a setting of eight quarter hectare enclosures in a luvic arenosol and with different population densities of common vole to take a snap shot on a set of important soil variables. Common voles (*Microtus arvalis* L.) and their *Arvicolinae* kin are naturally abundant and dig extensive burrow systems throughout the grassland and agricultural ecosystems of the northern hemisphere including Europe [7, 32, 33]. With a lifespan of about 18 months, an offspring of up to 40 per female yr^—1^, and significant decimation by predation, the population densities of common voles in open environments may reach up to about 215 individuals ha^–1^ [34, 35, 36]. Although during outbreaks, populations can increase to several thousand voles per hectare [37]. The impact of common voles on soils should go beyond the dislocation of soil matrix, because they carry their main diet of grasses and herbaceous plants to feed and defecate underground, thereby redistributing and concentrating nutrients [35].

We investigated four physical soil variables that control and/or characterize soil water relations (i.e., bulk density D_B_, infiltration rate IR, saturated hydraulic conductivity K_S_, and water holding capacity WHC). Another six variables were analyzed to detect effects of vole activity on the balance of soil carbon and nitrogen. The SOC and total N content were measured to estimate potential changes in their pools. Active and passive transport mechanisms can accelerate the changes in pools. The voles carry food and nesting material into their burrows and defecate. Similarly, rainwater drains much better through the burrows and can move litter, feces, and leached C and N compounds vertically through the soil column. The C/N ratio was calculated, because increased soil aeration and accelerated transport can shift the ratio at the various soil levels. Measurements of potential soil CO_2_ efflux were conducted to reflect changes in carbon mineralization, which can occur due to three mechanisms: (1) the breakup of soil aggregates and the exposing of old carbon to degradation, (2) a priming effect of enhanced carbon supply by, e.g. vole nesting, and enhanced soil microbial activity in lower compartments [38], and/or (3) increased soil ventilation and oxygenation through the voles’ burrow system. The natural abundance of isotopes in C (δ^13^C) and N (δ^15^N) were measured to serve as a proxy for processes linked to the C and N turnover [39, 40]. Furthermore, we determined the soil pH, which can change with both altered infiltration and leaching as well as altered soil nutrient status.

We hypothesized that (1) large differences in vole numbers and related bioturbation will cause differently accelerated changes in processes and pools of the measured soil variables in the upper 30-cm compartment, in which the animals lay out their burrow systems. (2) Hence, the enclosure experiment will allow quantification of vole effects on key biogeochemical cycles within a short 21-months period.

## Material and Methods

### Ethics statement

The experiment was conducted on land allocated to the University of Potsdam for research. The study did not involve endangered or protected species. Population founder animals were captured around Potsdam under permission of the Landesamt für Umwelt, Gesundheit und Verbraucherschutz Brandenburg (Referat Naturschutz; reference number RW-7.1 24.01.01.10). Animals, which were removed from the enclosures for population control, were released at the original trapping side of the population founder animals as specified in the experimental permission. The same state office approved also the enclosure experiments (Abteilung Verbraucherschutz; reference number V3-2347-44-2011). Teams were trained and took all possible precautions to minimize animal stress during trapping and sampling.

### Experimental setup

#### Field enclosures

Experimental plots were set up at a study site near Potsdam, State of Brandenburg, Germany [lat 52° 23’ N, lon 13° 3’ E] at a mean altitude of 35 m a.s.l. The area is part of the alluvial lowland, which drains waters in the East of Germany into the Baltic Sea. Mean annual precipitation and temperature for Potsdam is 600 mm and 8.7° C, respectively. Based on the World Reference Base for Soil Resources (WRB), the sandy soil is a luvic arenosol. Prior to the experiment, the land use was cropland for at least the last two centuries. At the times of agricultural production cooperatives, the plough depth was up to 60 cm. Soil explorations up to 100 cm within the plots showed transitions from the humus-enriched top soil to the sandy mineral soil at 30–40 cm depths, and further to a reddish clay-enriched (argillic) horizon at ≥70 cm depts.

**Fig 1:**
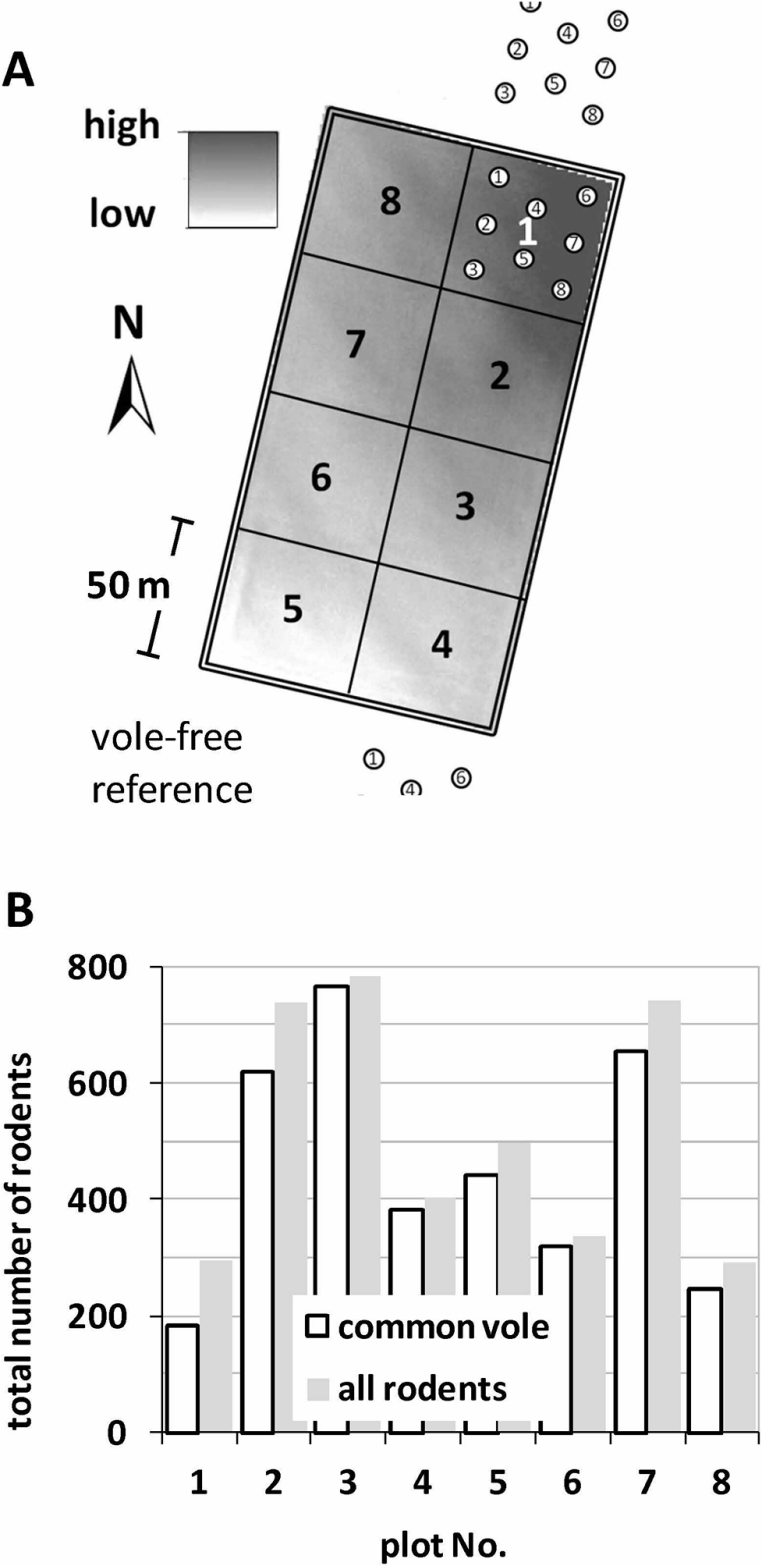
Experimental frame to study bioturbation effects on key soil biogeochemical variables. (A) The set up of plots #1–#8. A 0.3 m elevation gradient existed across the plot arrangement. Within-plot sample pattern for C and N variables is outlined in plot #1. Reference soil samples were collected from agricultural fields without vole populations north of plot #1 and south of plot #4. (B) 21 months total vole numbers per 2500-m^2^ plot. Very high and low vole numbers were achieved by repeated removal versus no removal of voles.

In 2009, eight 50×50-m plots were installed within an area of 200 m length and 100 m width (Fig 1A). Plot boundaries of steel plates reached 1 m into the soil, 0.5 m above the soil, and were equipped with electric fencing on top to fend off terrestrial mesopredators. There were no signs of burrows in the soil at the time of building the enclosures. Hence, it is a valid assumption that the pre-experimental vole population was negligibly small or nil.

The preferred diet of *Microtus arvalis* L. consists of grasses and forbs [17]. To provide appropriate nutritional value of forage for the development of vole populations [6], all plots received an initial seeding with grassland species including common bent (*Agrostis capillaris* L.), narrow leaved meadow grass (*Poa angustifolia* L.), meadow soft grass/velvet grass (*Holcus lanatus* L.), and sweet vernal grass (*Anthoxanthum odoratum* L.). Based on a subsequent vegetation survey listing 64 species in 2011, none of the sown species established well. The abundant species across plots were Italian ryegrass (*Lolium multiflorum* Lam.), annual bugloss (*Anchusa arvensis* L.), shepherd’s purse (*Capsella bursa-pastoris* (L.) Medik.), field poppy (*Papaver rhoeas* L.), dandelion (*Taraxacum officinale* Wigg), field violet (*Viola arvensis* Murray) and wheat (*Triticum spec*.). Further, strong growth of common nettle (*Urtica dioica* L.) developed within the SW-corner of plot #1. Tall-grown vegetation was cut twice a year. The biomass remained within the plots to maintain a similar food supply to voles, and to avoid large differences in C and N return across the enclosures.

Two features were not known at the outset of the study: (1) a post hoc made DEM from LiDAR data revealed a non-obvious 30-cm elevation gradient stretching along the NE–SW diagonal of the rectangle research site. (2) An archaeological survey prior to plot installation located a large hollow filled with brick fragments in the SW-corner of plot (#1) (Fig 1A).

#### Vole populations

The counting of voles was implemented by intermittent and final removal of animals. Intermittent removal served simultaneously for population control. The total number of rodents removed from all plots until the end of the experiment counted 4088, of which were 88.4% common vole (*Microtus arvalis* L), 8.1% harvest mouse (*Micromys minutus* Pallas), 2.5% striped field mouse (*Apodemus agrarius* Pallas) and only 0.1% bank vole (*Myodes glareolus* Schreber). The total number of common voles removed between July 2010 and March 2012 for plots #1 to #8 were: 184, 618, 767, 384, 443, 319, 653, and 246 (Fig 1B). Harvest mice and striped field mice entering the plots were not part of the experiment. However, as both do not dig burrows and are unlikely to contribute to the vertical redistribution of matter, their numbers were not included in calculating effects of bioturbation.

**Fig 2:**
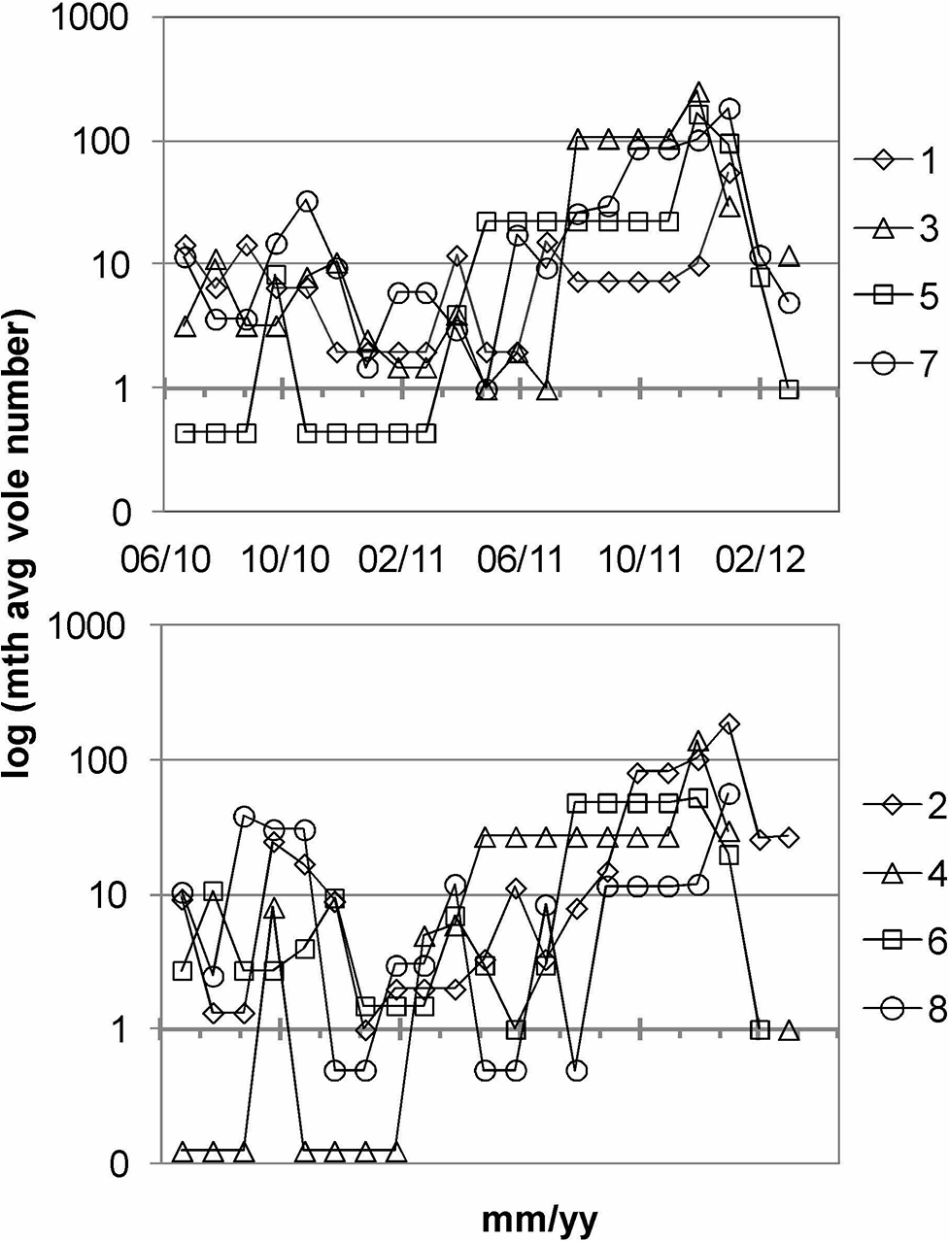
Development of vole populations within the eight plots based on repeated release, trapping and removal of voles between July 2010 and March 2012.

Common voles (always eight individuals plot^—1^) were released first in July 2010, except for plots #4 and #5, in which voles were released only in September 2010 (Fig 2). Population size was controlled by intermittently deploying Ugglan mice and vole traps (Ugglan special No2, Grahn AB, Sweden) with shrew-exits [41]. Voles were removed by the end of 2010, and later new founder animals were released to all plots. This measure provided an interim count for the first calendar year, it bypassed a part of the extreme cold period, it avoided inbred effects, and the new animals’ life span could theoretically outlive the experimental period. Hence, the sum of intermittent and final removal was prepared to record almost all voles that contributed to the 21-months bioturbation. No dead animals were found but losses to birds of prey could not be excluded.

Population control was stopped for the plots #3, #4, #5 and #7 by May 2011, while regular removal continued for the plots #1, #2, #6, and #8 until September 2011. Regular removal and vole count was resumed for all plots in November 2011 and continued until the end of the experiment. Vole populations were low to moderate in all plots in 2010; but they grew quickly without regular removal, and reached peak populations in fall 2011. Based on the assumption that vole bioturbation integrates over time, and in order to better reflect the varying number of voles, correlations were tested for the 21-months average number of voles in each plot and the biogeochemical variables.

### Measurements

Eleven key soil variables were sampled during field campaigns in March/April 2012. In each plot, eight sample locations for soil bulk density and C- and N-variables were spread in a pattern representing the area (3/2/3, counting from NW to SE in each plot, see Fig 1A). Hydraulic variables were sampled in a complementary pattern in between the former locations to avoid interferences from the different sampling. Soil bulk density and C- and N-variables but not the hydraulic variables were also sampled at vole-free reference locations to the south (C1) and north (C2) of the enclosed plots (Fig 1A).

Sampling depth was chosen with respect to the burrow systems of the common voles. Burrows consist of an aboveground runway network of 40 m, a tunnel system of about 15 m expanding in 10–20 cm depth in the root layer of plants, and a burrow network up to 40 cm depth, where voles have their nests and raise their young [33]. Accordingly, most of the sampled variables represented the upper 30-cm soil compartment. Different sets of sub-compartments were sampled to reflect appropriate spatial resolutions of variables (Table 1).

**Table 1:** Eleven biogeochemical key variables sampled from different soil compartments.

Soil variable	Unit	Sampling depths (cm)	Replicates/plot
Bulk density, D_B_	Mg m^–3^	0–15, 15–30	8
Infiltration rate, IR	cm min^–1^	surface	4
Saturated conductivity, K_S_	cm h^–1^	0–30	8
Water holding capacity, WHC	g g^–1^	0–15, 15–30	8
Soil organic carbon, SOC	kg m^–2^	0–5, 5–10, 10–20, 20–30	8
C stable isotope ratio (δ^13^C)		0–5, 5–10, 10–20, 20–30	8
Carbon dioxide efflux, CO_2_	μg g^–1^ soil s^–1^	0–15, 15–30	8
N content	kg m^–2^	0–5, 5–10, 10–20, 20–30	8
N stable isotope ratio (δ^15^N)		0–5, 5–10, 10–20, 20–30	8
C/N ratio			8
Soil pH		0–5, 5–10, 10–20, 20–30	8

Soil bulk density (D_B_) was determined as the ratio of the oven dry weight and the volume of a soil core taken with a 3-L steel core driver (height 150 mm, inner diameter 160 mm). Infiltration rate was measured using a double ring infiltrometer (Eijkelkamp, NL) pursuant to DIN 19682–7 [42]. Saturated hydraulic conductivity (K_S_) was examined using a constant-head permeameter (KSAT Inc., Raleigh, NC, USA) according to the methodical descriptions of Amoozegar [43]. Water holding capacity was determined in the form of container capacity [44] referring to the water-to-soil weight ratio in a small soil sample (2-mm sieved soil in 0.2-L steel cylinder) after 24 h on a wet sand surface.

Potential CO_2_ efflux was quantified one and two weeks after 300 g samples of 2-mm sieved soil from the D_B_ samples were incubated at 25°C and 40% WHC in a climate chamber. Measurements were conducted using an automatic system [45–47], which connects consecutively 48 dynamic incubation chambers (and an ambient air offset correction) to C stable isotope sensitive wavelength scan cavity ring-down spectroscopy (WS-CRDS, model G1101-i, Picarro, CA, USA). Total net CO_2_ efflux from soil samples was obtained from the sum of the individual ^12^CO_2_ and ^13^CO_2_ fluxes.

For the analysis of soil organic carbon content (SOC), soil nitrogen content (N), and the C and N stable isotope ratios (δ^13^C and δ^15^N), additional 300-g soil cores were taken side by side with the D_B_ samples (for the sampled depths see Table 1). SOC, N, δ^13^C and δ^15^N were measured using an Elemental analyzer (EA, Vario EL III elemental analyzer [CHNOS], Elementar, Hanau, Germany) coupled via a Conflow-III interface (Thermo Electron Group, Waltham, Massachusetts, USA) to a continuous flow isotope ratio mass spectrometer (IRMS, Delta V Plus, Thermo Fisher Scientific, Bremen, Germany). Routine EA-IRMS analysis included samples containing carbonate (i.e. inorganic C) for nitrogen analysis and samples, from which carbonate was catalytically removed for organic carbon analysis. The procedure allowed obtaining correct SOC and δ^13^C values, and it avoided bias of acidification on N contents and δ^15^N values [48].

Soil pH of 10-g dried and sieved soil samples was determined in 0.01 M CaCl_2_ solution using a pH Meter (Microprocessor pH-mV Meter pH 526, WTW, Weilheim, Germany).

### Statistical analysis

Statistical analysis was performed using SPSS 20 (SPSS Inc., Chicago, USA). None of the cores for sampling C and N related data met immediately with a vole tunnel. All C and N related data passed an outlier test (threshold three standard deviations based on the total sample). The outlier test was not applied to water related data (K_S_ and IR), because adjacent tunnels can be expected to produce large differences. Soil variables were tested for Pearson correlation (two-tailed, r, coefficient range -1 to 1) with the 21-months average number of voles. Visual analysis revealed an interference of the small elevation gradient. Hence, all variables were subjected to a multiple regression with average number of voles and the elevation gradient as independent variables (summary in Table 2). Correlations indicate significant (sign.*, 0.05 level), highly significant (sign.**, 0.01 level), and not significant (sign. ≥0.05). Individual Pearson correlations are quoted in the text for variables that did not correlate with elevation. The R^2^ in the last two figures shows the coefficient of determination of linear regression (range 0 to 1) to visualize possible relationships.

**Table 2:** Results of the multiple regression with average number of voles (avnv) and elevation (elev) as independent variables.

	Significance	r	Pearson coefficients	
	avnv	elev	avvn	elev
K_S_	0.456	0.000**		0.661
IR	0.074	0.216		
D_B__15	0.405	0.212		
D_B__30	0.164	0.267		
WHC_15	0.215	0.215		
WHC_30	0.244	0.022*		-0.258
CO_2__15	0.077	0.003**		-0.342
CO_2__30	0.095	0.013*		-0.277
δ^13^C_30	0.342	0.093		
δ^15^N_30	0.013*	0.148	0.279	
SOC_30	0.138	0.000**		-0.438
N_30	0.326	0.001**		-0.377
C/N_30	0.155	0.009**		-0.296
pH_30	0.421	0.000**		-0.583
δ^13^C_20	0.471	0.29		
δ^15^N_20	0.000**	0.000**	-0.464	-0.652
SOC_20	0.152	0.000**		-0.564
N_20	0.221	0.000**		-0.617
C/N_20	0.147	0.235		
pH_20	0.373	0.000**		-0.568
δ^13^C_10	0.438	0.053		
δ^15^N_10	0.103	0.015*		-0.281
SOC_10	0.089	0.000**		-0.566
N_10	0.219	0.000**		-0.635
C/N_10	0.001**	0.026*	-0.401	0.253
pH_10	0.444	0.000**		-0.59
δ^13^C_5	0.374	0.377		
δ^15^N_5	0.342	0.322		
SOC_5	0.137	0.000**		-0.445
N_5	0.295	0.000**		-0.529
C/N_5	0.147	0.432		
pH_5	0.305	0.000**		-0.624

Correlations are significant at the 0.05 level (*) and highly significant at the 0.01 level (**). The Pearson coefficients are shown only for significant correlations. Variable abbreviation as in Table 1; numbers to the abbreviations indicate the lower depth of the compartment.

## Results

**Fig 3:**
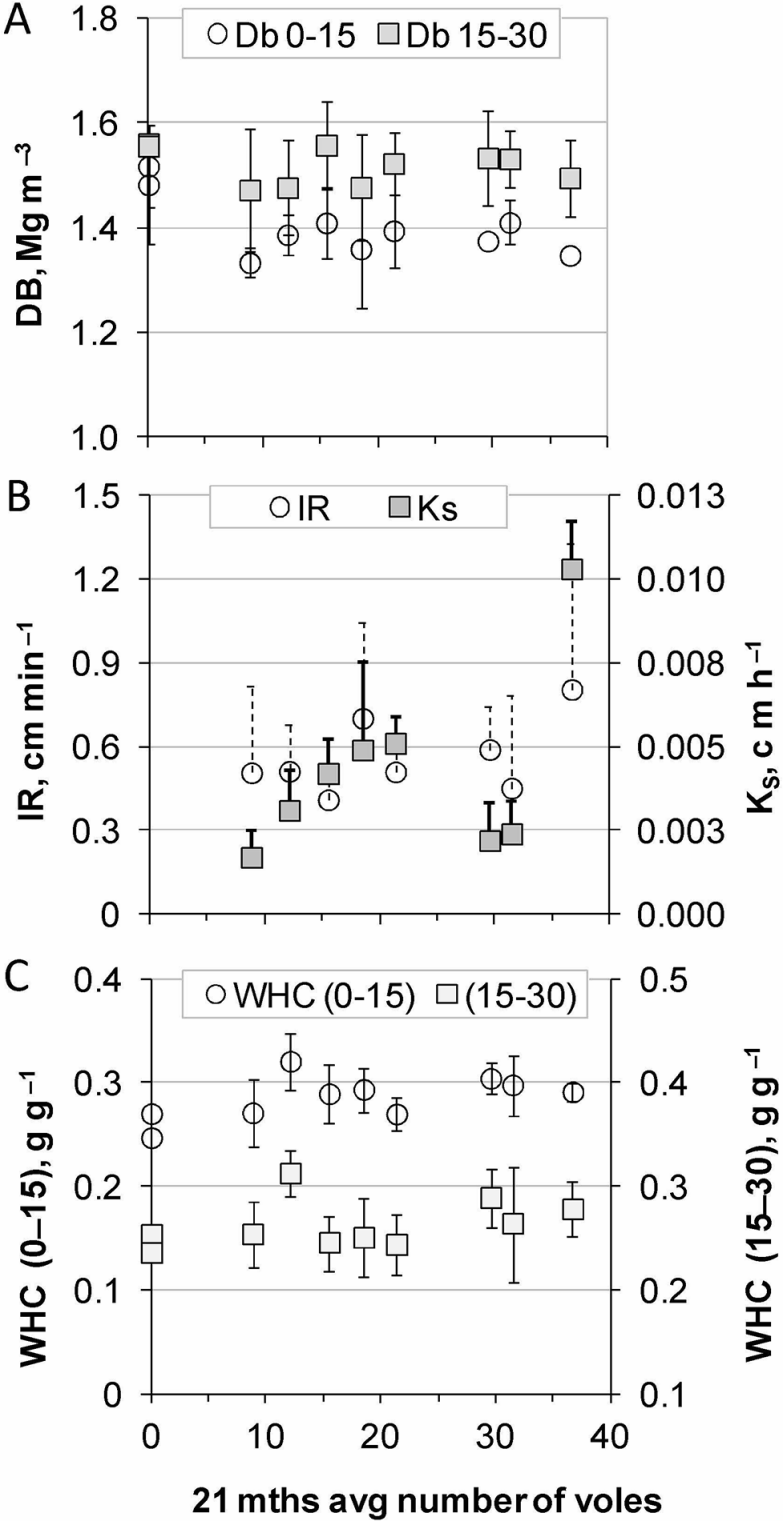
(A) Soil bulk density (D_B_), (B) infiltration rate (IR) and saturated hydraulic conductivity (Ks), and (C) water holding capacity (WHC) with increasing number of voles per plot. Mean and SD of soil variables, in (B) only one-sided SD.

Bioturbation can be first visualized as the displacement and disturbance of a settled soil matrix. Mean soil bulk density varied across all plots between 1.33 and 1.52 Mg m^–3^ and throughout higher values of 1.46 and 1.56 Mg m^–3^ for the 0–15 cm and 15–30 cm compartment, respectively. Bulk density seemed unaffected by vole numbers (0–15 cm sign. = 0.81, 15–30 cm sign. = 0.33), and at least three plots with much less voles than the crowded plot #3 showed comparably low bulk density values (Fig 3A). Plot #3 included a monthly average of 36.5 voles and a peak density of 252 voles per plot in December 2011 (equivalent to 1008 voles ha^–1^).

Similarly, infiltration rate (IR) and saturated hydraulic conductivity (K_S_) did not exhibit significant correlations with vole numbers (Table 2), although, the corresponding lowest and highest value pairs contributed in either case strongly to a trend of increasing IR and K_S_ with increasing number of voles (Fig 3B). Notably, K_S_ was one of the eight key variables that correlated with the elevation gradient across the plot arrangement (Table 2).

Laboratory based soil water holding capacity (WHC) varied between 0.25–0.32 g g^—1^ and 0.24–0.31 g g^—1^ for the upper (0–15 cm) and lower (15–30 cm) soil compartment, respectively (Fig 3C). WHC did not show any trend with respect to vole numbers (0–15 cm sign. = 0.43) but the lower compartment showed a significant correlation with the elevation gradient (WHC 15–30 cm, Table 2). Furthermore, excluding plot #1, the overall WHC (0–30 cm) of plots correlated positively with the averaged SOC content (0–30 cm) (sign.* = 0.016; linear regression R^2^ = 0.72, WHC = 0.11 SOC + 0.14). Plot #1, which was at the highest elevation and included the filled hollow in one corner, had by far the largest total SOC content but a rather low WHC among all plots.

**Fig 4:**
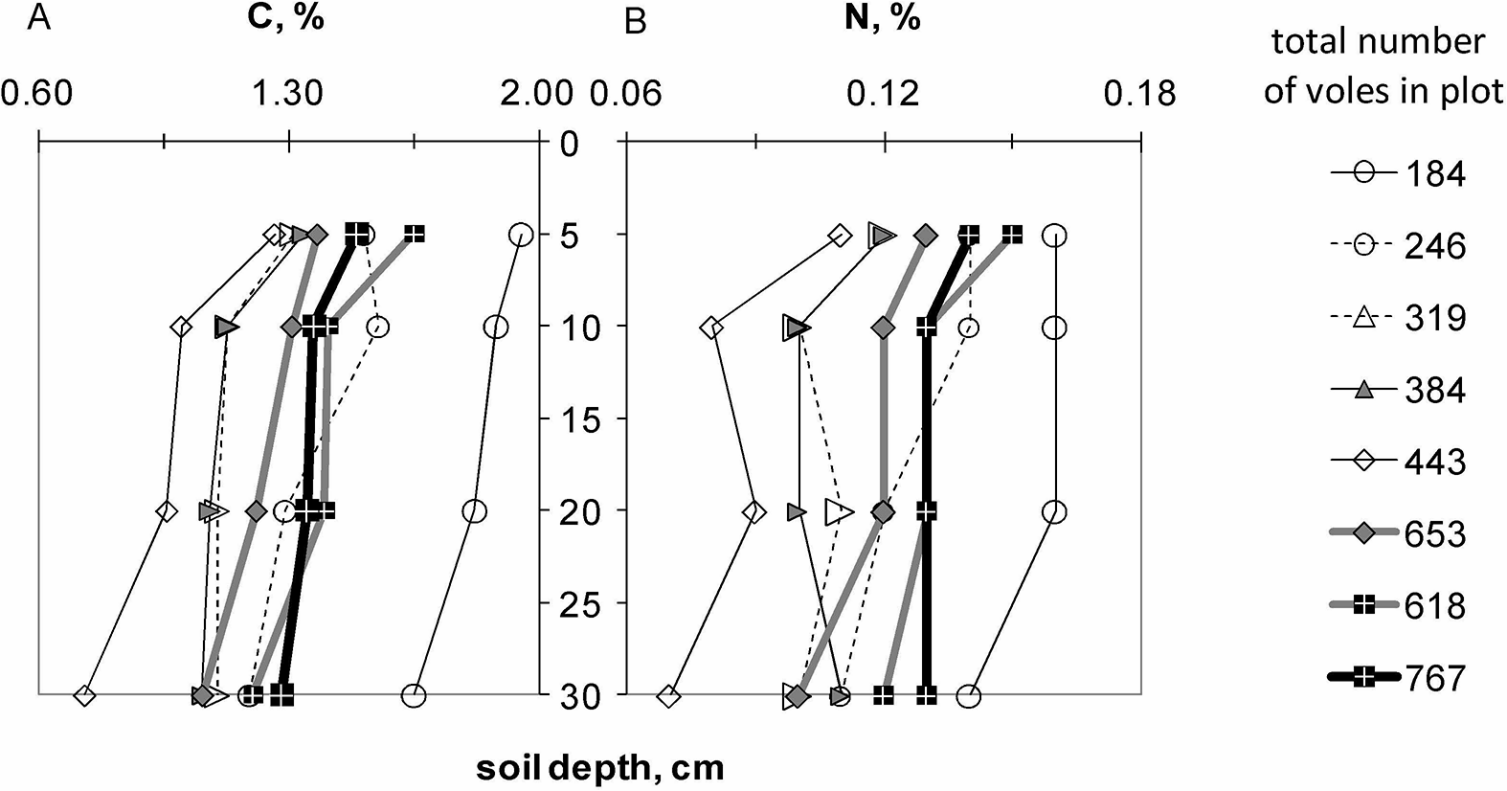
Vertical variability of (A) soil organic carbon content and (B) N contents at different numbers of voles.

The total SOC stocks in the upper 30 cm ranged between 1.4–2.5 kg C m^–2^ with a mean across plots of 1.88 kg C m^–2^. SOC decreased from the highest elevation plot (#1) to the lowest elevation plot (#5) and all sampled layers showed highly significant correlations to the elevation gradient across plots (r = -0.438–-0.566, sign. Table 2). SOC stocks of the individually examined soil layers at the Potsdam plots did not correlate with vole numbers (sign.* ≥ 0.09). SOC content decreased generally with soil depth. With respect to the vertical changes in SOC and similarly N contents across plots, it first appeared that increasing number of voles led to increasingly homogeneous amounts of SOC and N across the soil layers (Fig 4A and 4B). However, the correlation between the total variances in SOC or N across depths and the vole numbers were not significant (C r = -0.51, sign. = 0.19, N r = -0.42, sign. = 0.31).

**Fig 5:**
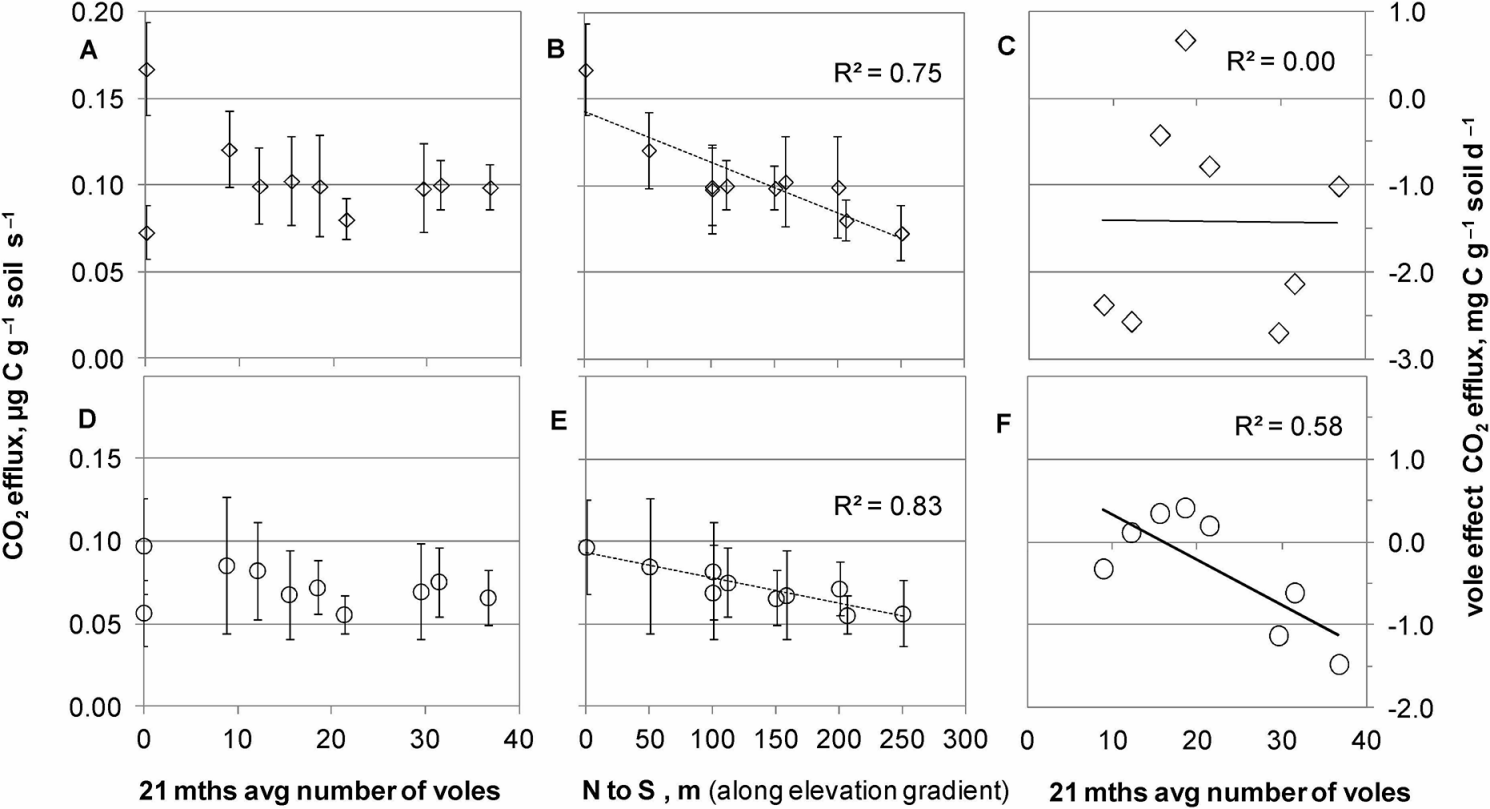
Soil CO_2_ efflux and vole effects. Soil CO_**2**_ efflux (left Y-axis) from the 0–15 cm (A, B) and the 15–30 cm compartment (D, E) versus (A, D) increasing numbers of voles, and (B, E) the elevation gradient from N (0 m) to S (250 m) (with 0 and 250 representing the reference samples outside the vole plots). (C, F) show the voles’ net effect (right Y-axis) on the CO_**2**_ efflux from the (C) 0–15 cm and (F) the 15–30 cm compartments.

The net CO_2_ efflux from the samples of the different plots did not correlate with the 21-months average number of voles (Fig 5A and 5D) but generally with the elevation gradient (Fig 5B and 5E). However, this changed for the lower of the investigated compartments when data were baseline corrected (15–30 cm corrected, sign.* = 0.029) eliminating the elevation effect that resulted from the different soil core potential CO_2_ efflux of the northern and southern field reference samples (Fig 5E). The vole effect on the potential CO_2_ efflux decreased by 5.4 mg C g^—1^ soil day^—1^ per 100 voles in a plot (see regression in Fig 5F: about 1 unit per 20 voles).

**Fig 6:**
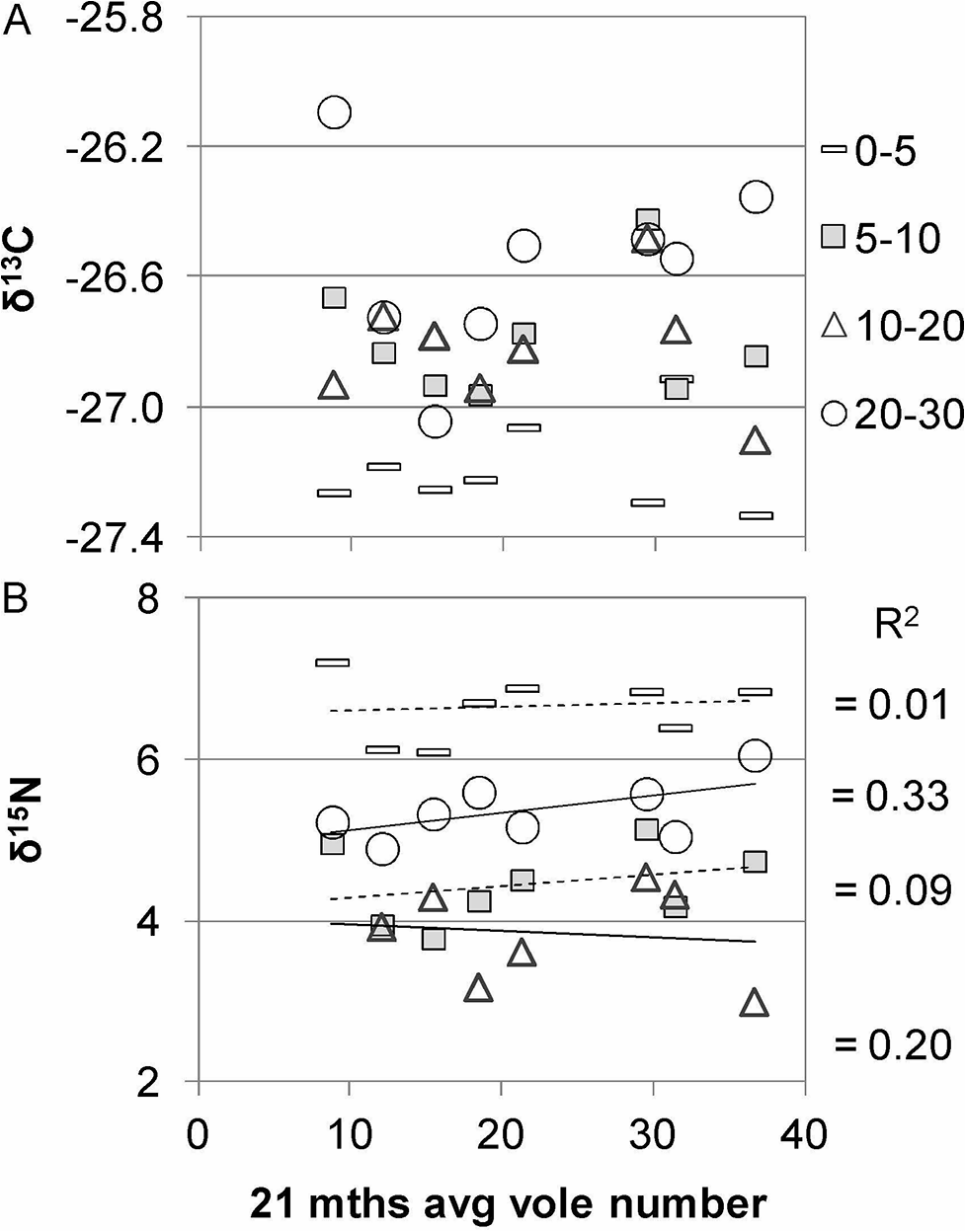
Changes in the stable isotope ratios of (A) carbon and (B) nitrogen in four soil layers versus increasing vole numbers. The soil depth signature is the same in both charts. R^2^ values correspond to the nearest signatures.

The δ^13^C differed between -26.25‰ and -27.33‰ in the layers from 0–30 cm with a slight increase towards deeper soil. Interestingly, the pattern of δ^13^C values across plots and soil layers did not reveal any influence of the elevation gradient or the vole numbers on soil carbon mineralization processes (Fig 6A, Table 2).

Mean soil N stocks (0–30 cm) across plots ranged between 0.13–0.22 kg N m^–2^. Similar to the SOC, the soil nitrogen contents (N, %) showed (1) a clear correlation to plot elevation, (2) no correlation of individual soil layers N to vole numbers (Table 2), and (3) a slightly leveling influence of voles on the variability of N contents across layers (Fig 4B).

The C/N ratio varied between 9.78–19.59 across plots and soil layers. The ratios did not correlate with elevation or vole numbers in the soil layers 0–5 cm and 10–20 cm. The C/N ratio showed a strong negative correlation with the elevation gradient in the lowest layer (20–30 cm, r = -438, sign.** <0.001). In contrast, the C/N ratio at 5–10 cm was positively correlated with elevation (r = 0.253, sign.* = 0.026) and showed a highly significant negative correlation with vole numbers (r = -0.401, sign.** <0.0008). The latter significance improved slightly with the total number of rodents replacing the average vole number (sign.** < 0.0005).

The δ^15^N values of the upper two soil layers were not significantly influenced by voles (sign. ≥ 0.1). But the 5–10 cm layer did correlate with elevation. However, the δ^15^N values of the 10–20 cm and the 20–30 cm compartments showed a highly significant negative correlation (r = -0.464, sign.** ≤ 0.001) and significant positive correlation (r = 0.279, sign.* = 0.013 with vole numbers, respectively (Fig 6B). Intriguingly, δ^15^N at 10–20 cm still correlated similarly with the elevation (Table 2).

We checked with small pools and process indicators whether the occurrence of “other rodents” might have influenced other bioturbation result. Neither δ^15^N nor the N stocks showed correlations with the total number of rodents. The δ^15^N of the uppermost compartment (0–5 cm), i.e., probably the most sensitive case, did also not indicate a correlation (sign. = 0.45).

The pH varied between 6 and 7.5 across plots and soil layers and correlated strongly with elevation but not with vole numbers.

The available data basis of soil biogeochemical variables is summarized in S1 Table. For the exact interpretation of vole effects, it must be noted that the pattern of significantly and not-significantly correlated variables did not change, when they were based only on the vole numbers from November 2011 to March 2012, i.e., the final period emphasizing the development of the population peaks.

## Discussion

### Vole effects on soil variables

Based on eight grassland plots with different vole densities, we assumed that vole activity affects soil characteristics of the upper 30-cm compartment, in which the animals lay out their burrow systems. After 21 months of vole activity, and three to four months after the peak densities of voles were measured, seven out of 11 key variables did not show correlations with vole population density.

The mean SOC and N contents of the upper 30-cm compartment across the vole plots were only slightly lower than the average for the Arenosols of Central and Eastern Europe (plot means vs. Central Europe average, kg m^–2^: SOC 1.9 vs 2.2, N 0.17 vs. 0.21 (see [49]). The similar applied for the soils’ C/N ratio (11.4 vs. 11.6). SOC and N pools in the Potsdam plots seemed not affected by increasing vole numbers after 21 months. This is less surprising because pools of SOC and N do not change quickly as priming effects, increased mineralization, volatilization and leaching control the disposition of increased inputs [38, 50, 51]. Still, all of these dynamics are likely enhanced through the burrowing activity of voles over longer time periods. Manaeva et al. [52] found seasonal changes in the C and N contents of an Agrozem inhabited by southern voles as compared to a vole-free area at the Botanical Garden of Moscow State University. However, the study samples were “collected from the paths and different hole chambers of the rodents”, and thus, they seemed to have immediate contact with the rodents and their excretions. Gervais et al. [27] report that extractable SOC, SOM, soil NH_4_^+^ and NO_3_^—^values were larger immediately below vole tunnels than above, but their results were not significant due to small sample sizes. Villarreal et al. [53] found significant increases in N at burrow plots compared to non-burrow plots of *Lagostomus maximus* (family Chinchillidae) in La Pampa Province, Argentina. However, 40% of the soil cores taken in the colonized plots met with burrows, whereas none of the soil cores sampled at the Potsdam plots had such immediate contact.

Generally, SOC and N contents of the Potsdam plots decreased with depth. In connection to the core feature of bioturbation, one might expect that vole activity fills SOC and N pools of lower and depleted compartments [54], and thereby tends to homogenize the respective values across soil layers. Indeed, the plot with the lowest vole number showed by far the largest total variance in SOC and N content, but the tendency was not corroborated, as increasing vole numbers did not produce consistently lower variances.

Estimates of vole effects on the N cycle in particular will be further impeded, because the combined feedbacks of voles’ alteration of vegetation and litter, and the fluctuations in vole populations itself, can prevent the development of linear effects through time [31]. All this puts more weight on the most sensitive variables of the natural abundance of C and N stable isotopes. Decreasing and increasing δ^15^N with vole numbers indicate lower mineralization at the 10–20 cm level and higher mineralization at the 20–30 cm level, respectively. Increasing δ^15^N values indicate higher N cycle process rates and higher N loss [39]. Hence, increasing δ^15^N values with increasing vole counts may be related to nutrient concentration in soil due to fecal inputs and nesting activities with subsequently enhanced N mineralization rates. Enhanced N mineralization involves larger N gas losses due to nitrification and denitrification, as well as larger leaching following precipitation [39]. Vole feces are quickly mineralized due to their small grain and large surface with fecal N residence times of few days [25]. In the present study, the quick vole-induced changes in δ^15^N may have been facilitated by the low total soil N content so that the increased δ^15^N signal of fractionation-affected N process source pools quickly became significant against the low total soil N background. Overall, vole-induced higher nutrient releases to nutrient-poor soils would probably draw a soon response of vegetation composition.

Potential soil CO_2_ emission from samples of the lower compartment decreased with increasing vole activity in 15–30 cm depth, but the same flux remained unaffected by vole density in the 0–15 cm uppermost topsoil. Consequently, it appears that voles decreased soil C mineralization mostly in their major burrow level. This could be due to a depletion of the more labile carbon fractions by increased C mineralization throughout the 21 months the soils were exposed to vole activity. In contrast to our study, Manaeva et al. [52] found increased soil CO_2_ production of a soddy podzolic soil from the lower burrow level of bank voles at the Chernogolovka experimental base, Russia. The soil and climatic conditions were very different between the Potsdam plots and the Chernogolovka experimental base, and we lack details on the persistence of the vole populations. Most importantly however, the immediate proximity of collected samples to vole activity may provide less information pertaining to the voles’ effect on the bulk soil compartment. Increased mineralization can be caused by increased aeration through the vole tunnels and priming of native SOC upon repeated availability of fresh organic matter. This process would mainly affect the compartment in which the tunnels expand but less the upper compartment, which forms the roofing of the tunnel system.

In contrast, decreased CO_2_ emission from the soil could be due to a positive effect of voles on SOC stabilization. It has been reported that burying activity of the soil biota affects the incorporation of organic carbon into mineral-organic aggregates [55], and thus, protecting organic carbon from decomposition [56]. It has been postulated that the incorporation of organic matter into aggregates and in deeper soil layers has an overall positive effect on SOC storage [57, 58]. Overall, the contrasting findings point to more research being needed for a consistent understanding of the interactions of environmental conditions and process mechanisms that control bioturbation effects on soil respiration.

Soil bulk density (D_B_) affects soil aeration, water relations and plant growth, and via these cross links also soil biogeochemistry; root growth by itself can change D_B_. The current results did not show any influence of voles on the D_B_ of a sandy luvic arenosol. A population of 500 voles might reduce a D_B_ of 1.5 Mg m^–3^ by less than 0.1 Mg m^–3^ within a 2500-m^2^ plot, if (1) every vole digs a tunnel of 0.03 m inner diameter and 15 m length [33], and (2) if all tunnels collapse. When tunnels collapse, new tunnels are dug, but the small rodents deposit tailings also in abandoned tunnels [14]. Hence, belowground compression of soil to stabilize tunnels and chambers may also occur, and intact burrow systems should not change the D_B_ of the remaining soil compartment. Furthermore, the sandy soil in combination with runoff fairly restricted through plot walls may have also enhanced re-sedimentation. So we revisit the question, to which extent can collapsed tunnels and spreading of solid discharge on the soil surface can contribute to a lower D_B_ of a sandy soil within two years? From our data it seems, the chances to find a change in D_B_ with eight 16-cm soil cores within a quarter ha plot were, at best, to hit an intact tunnel.

The examination of WHC in the form of container capacity turned out being a low-key variable because it does not reflect the in-situ soil compactness and depends exclusively on the voles’ capacity to significantly change SOC contents. Instead, the elevation gradient across the land still affected SOC content and thereby the WHC.

In situ infiltration rate and saturated hydraulic conductivity could have been more sensitive than D_B_ in terms of reflecting a somewhat larger portion of the soil compartment and its short term history. Both variables pointed to a marginal trend to increase with increasing vole numbers, which was in neither case statistically significant.

Laundré [59] found a higher soil water recharge in rangeland plots with small mammal burrows than in non-burrow plots. The soils of those plots in Idaho seemed to contain more silt and loam than at our plots, and more importantly, higher recharge was only observed in dry years and not in wet years. Zaitlin et al. [24] could also not confirm clear effects of burrowing activity on infiltration rates in a haplic chernozem within the prairie pothole region in Canada. Zaitlin and Hayashi [60] conclude that burrowing mammals generally increase the patchiness of an environment. Overall, this suggests that the soil memory for physical bioturbation effects is spatially and temporally too diverse as to expect statistically significant results from a number of point samples. It further suggests that neither time nor plot makes a difference, but the better solution to quantify the influence of burrowing rodents on soil water relations may lie with large scale exclusion experiments in a flat terrain, which can be monitored by remote sensing to reveal the dynamics of surface wetness.

### Relation of vole density to soil variables

Interrelating values of vole numbers and soil variables does not necessarily compare one with other time-integrated results. Vole populations go through regular multi-annual as well intra-annual density fluctuations [61, 62]. Especially in microtine voles, large winter population declines have been reported [63–65]. Birds of prey and mammalian mesopredators regulate vole populations in grasslands and agricultural landscapes [66]; around human habitation, domestic cats can exert significant predation pressure [67]. Thus, predominantly climate, food supply, and predation interact in controlling vole populations. Particularly, the two latter factors may cause even larger fluctuations in severely disconnected patches such as the ones represented by the tin-cased field plots [68]. The set up restricted the voles from escaping, and reduced the chance of terrestrial mesopredators to scavenge from the plots. Furthermore, vegetation growing at times up to breast height seemed to limit the plot activity of birds of prey. Vole populations ten times higher than in the open range let us assume that their development was largely decoupled from predation, which otherwise would have caused fluctuations in vole densities [69, 70] not correlated with control measures. The essence of the above excursion into vole population dynamics is that we cannot derive and extrapolate exact vole power hours per year, because of the limited experimental control on vole numbers in extra large mesocosms. Finally, even for vole populations developing according to a standard growth curve, we still lack the information, how burrow systems expand with increasing vole numbers. A detailed study on their home ranges suggested not every animal builds its structures but burrow systems are widely shared as a result of the social organization of *Microtus* voles in dense, kin-clustering colonies [71]. This aspect is corroborated by the observation that voles maintain the burrow networks over generations [27].

With the various constraints in mind, rather a two-hundred than a hundred-individual difference in vole count may be the order of threshold for the proper resolution of bioturbation effects on soil variables. Hence, we may expect, that short term experiments of, e.g., 21 months allow detection but not exact quantification of bioturbation effects. On the other hand, the peak populations may have brought their plots close to an ecological meltdown [72], therefore certainly raising concerns about the rational longevity of such experiments. Even experiments with moderate vole populations may over the long term change the vegetation and incorporate more indirect effects such as a changing C/N ratio [31]. The response of the C/N ratio in the 5–10 cm layer may be due to surface N-deposition by “all rodents” and a quick percolation with spring precipitation. As for the change in vegetation, it seemed remarkable that the plots contained diverse herbaceous biomass and less grasses at the time of soil variable sampling.

At the end, it did not escape our notice that some data disagreed with the assumption of “linear effects with increasing vole numbers”. Looking closely at, e.g. the δ^13^C (Fig 6A), the pattern might indicate a decrease followed by an increase or vice versa. Variably fast and interacting processes must not necessarily involve unidirectional changes. If the change in a variable is the discontinuous function of multiple factors of varying strength, the resolution of our data was not sufficient to show such pattern clearly. Future snapshots may involve new queries but will probably answer some of the questions presently remaining.

## Conclusions

The soil variables responding and not responding to vole density within two years seem to group mainly into those sensitive to changes in dynamics (CO_2_ efflux and δ^15^N) and those sensitive to—in the broader sense—changes in pools, respectively. This result complies with the concept that changes must first be established in dynamics before significant changes in pools can be expected. Also the δ^13^C result fits this line of thought, because the SOC pools are much larger and soils are usually more limited in N than in C. However, as dynamics start to change within just 21 months, we may expect the pools to follow with a few years of delay. Essentially, the results outline (1) the relative resilience of temperate soils to short term bioturbation by voles, and (2) that quantitative results of vole effects may be obtained from this type of enclosure experiments, if they are repeated over some (5–10) years.

## Supporting Information

S1 Table Plot data of the eleven biogeochemical soil variables (mean and standard deviation sd, n = 8, except IR n = 4). (DOC)
