# Supplementary material for: Effects of Short Term Bioturbation by Common Voles on Biogeochemical Soil Variables
Source: PLoS One. 2015 May 8;10(5):e0126011. doi: 10.1371/journal.pone.0126011 (PMC4425692; doi:10.1371/journal.pone.0126011)
Supplement: S1 Table — (DOC) [file pone.0126011.s001.doc]

Variables unit

| K_S_ | Saturated hydraulic conductivity | cm h^-1^ | | |  | |
| --- | --- | --- | --- | --- | --- | --- |
| IR | Infiltration rate | cm min^-1^ |  |  | |  |
| DB_15 | Bulk density 0 to 0.15 m | Mg m^-3^ |  |  | |  |
| DB_30 | Bulk density 0.15 to 0.3 m | Mg m^-3^ |  |  | |  |
| WHC_15 | Water holding capacity 0 to 0.15 m | g g^-1^ (soil dry weight) |  |  | |  |
| WHC_30 | Water holding capacity 0.15 to 0.3 m | g g^-1^ (soil dry weight) |  |  | |  |
| SOC_5 | SOC content 0 to 0.05 m | % |  |  | |  |
| SOC_10 | SOC content 0.05 to 0.1 m | % |  |  | |  |
| SOC_20 | SOC content 0.1 to 0.2 m | % |  |  | |  |
| SOC_30 | SOC content 0.2 to 0.3 m | % |  |  | |  |
| CO2_15 | CO2 emission potential 0 to 0.15 m | µg C g^-1^ s^-1^ (soil dry weight) |  |  | |  |
| CO2_30 | CO2 emission potential 0.15 to 0.3 m | µg C g^-1^ s^-1^ (soil dry weight) |  |  | |  |
| d13C_5 | Carbon stable isotope ratio 0 to 0.05 m | ‰ |  |  | |  |
| d13C_10 | Carbon stable isotope ratio 0.05 to 0.1 m | ‰ |  |  | |  |
| d13C_20 | Carbon stable isotope ratio 0.1 to 0.2 m | ‰ |  |  | |  |
| d13C_30 | Carbon stable isotope ratio 0.2 to 0.3 m | ‰ |  |  | |  |
| N_5 | Soil nitrogen content 0 to 0.05 m | % |  |  | |  |
| N_10 | Soil nitrogen content 0.05 to 0.1 m | % |  |  | |  |
| N_20 | Soil nitrogen content 0.1 to 0.2 m | % |  |  | |  |
| N_30 | Soil nitrogen content 0.2 to 0.3 m | % |  |  | |  |
| d15N_5 | Nitrogen stable isotope ratio 0 to 0.05 m | ‰ |  |  | |  |
| d15N_10 | Nitrogen stable isotope ratio 0.05 to 0.1 m | ‰ |  |  | |  |
| d15N_20 | Nitrogen stable isotope ratio 0.1 to 0.2 m | ‰ |  |  | |  |
| d15N_30 | Nitrogen stable isotope ratio 0.2 to 0.3 m | ‰ |  |  | |  |
| C/N_5 | C/N ratio 0 to 0.05 m |  |  |  | |  |
| C/N_10 | C/N ratio 0.05 to 0.1 m |  |  |  | |  |
| C/N_20 | C/N ratio 0.1 to 0.2 m |  |  |  | |  |
| C/N_30 | C/N ratio 0.2 to 0.3 m |  |  |  | |  |
| pH_5 | pH 0 to 0.05 m |  |  |  | |  |
| pH_10 | pH 0.05 to 0.1 m |  |  |  | |  |
| pH_20 | pH 0.1 to 0.2 m |  |  |  | |  |
| pH_30 | pH 0.2 to 0.3 m |  |  |  | |  |

| Plot | 1 | sd | 2 | sd | 3 | sd | 4 | sd |  |  |  |  |  | sd | 8 | sd |
| --- | --- | --- | --- | --- | --- | --- | --- | --- | --- | --- | --- | --- | --- | --- | --- | --- |
| K_S_ | 1.74E-03 | 8.18E-04 | 2.20E-03 | 9.40E-04 | 3.79E-03 | 1.39E-03 | 4.97E-03 | 2.61E-03 |  |  |  |  |  | 1.12E-03 | 3.18E-03 | 1.19E-03 |
| IR | 5.06E-01 | 2.66E-01 | 5.88E-01 | 2.84E-01 | 8.00E-01 | 4.49E-01 | 7.00E-01 | 2.96E-01 |  |  |  |  |  | 1.33E-01 | 5.13E-01 | 1.43E-01 |
| DB_15 | 1.33E+00 | 1.15E-01 | 1.37E+00 | 6.98E-02 | 1.35E+00 | 4.22E-02 | 1.36E+00 | 3.85E-02 |  |  |  |  |  | 1.13E-01 | 1.39E+00 | 7.97E-02 |
| DB_30 | 1.47E+00 | 1.16E-01 | 1.53E+00 | 9.16E-02 | 1.50E+00 | 7.32E-02 | 1.48E+00 | 1.01E-01 |  |  |  |  |  | 5.39E-02 | 1.48E+00 | 9.12E-02 |
| WHC_15 | 2.71E-01 | 3.30E-02 | 3.04E-01 | 1.48E-02 | 2.91E-01 | 6.81E-03 | 2.93E-01 | 2.08E-02 |  |  |  |  |  | 1.94E-02 | 3.17E-01 | 2.59E-02 |
| WHC_30 | 2.54E-01 | 3.27E-02 | 2.89E-01 | 2.83E-02 | 2.78E-01 | 2.70E-02 | 2.50E-01 | 3.85E-02 |  |  |  |  |  | 5.46E-02 | 3.16E-01 | 2.32E-02 |
| SOC_5 | 1.95E+00 | 8.10E-01 | 1.65E+00 | 4.63E-01 | 1.49E+00 | 1.62E-01 | 1.34E+00 | 1.40E-01 |  |  |  |  |  | 2.17E-01 | 1.51E+00 | 2.35E-01 |
| SOC_10 | 1.88E+00 | 8.03E-01 | 1.41E+00 | 3.02E-01 | 1.37E+00 | 1.85E-01 | 1.14E+00 | 1.98E-01 |  |  |  |  |  | 1.94E-01 | 1.55E+00 | 4.73E-01 |
| SOC_20 | 1.82E+00 | 7.09E-01 | 1.40E+00 | 3.18E-01 | 1.35E+00 | 1.83E-01 | 1.08E+00 | 2.00E-01 |  |  |  |  |  | 1.83E-01 | 1.29E+00 | 1.81E-01 |
| SOC_30 | 1.65E+00 | 8.27E-01 | 1.20E+00 | 4.11E-01 | 1.28E+00 | 2.35E-01 | 1.06E+00 | 3.02E-01 |  |  |  |  |  | 3.44E-01 | 1.19E+00 | 3.40E-01 |
| CO2_15 | 1.21E-01 | 2.20E-02 | 9.82E-02 | 2.55E-02 | 9.89E-02 | 1.26E-02 | 9.95E-02 | 2.92E-02 |  |  |  |  |  | 1.41E-02 | 9.97E-02 | 2.23E-02 |
| CO2_30 | 8.52E-02 | 4.11E-02 | 6.94E-02 | 2.87E-02 | 6.59E-02 | 1.67E-02 | 7.18E-02 | 1.62E-02 |  |  |  |  |  | 2.07E-02 | 8.21E-02 | 2.93E-02 |
| d13C_5 | -2.73E+01 | 3.46E-01 | -2.73E+01 | 2.11E-01 | -2.73E+01 | 4.78E-01 | -2.72E+01 | 2.88E-01 |  |  |  |  |  | 2.55E-01 | -2.72E+01 | 3.40E-01 |
| d13C_10 | -2.67E+01 | 2.85E-01 | -2.64E+01 | 4.16E-01 | -2.68E+01 | 6.30E-01 | -2.70E+01 | 3.28E-01 |  |  |  |  |  | 4.54E-01 | -2.68E+01 | 3.61E-01 |
| d13C_20 | -2.69E+01 | 4.87E-01 | -2.65E+01 | 1.34E+00 | -2.71E+01 | 7.48E-01 | -2.69E+01 | 1.59E-01 |  |  |  |  |  | 6.59E-01 | -2.67E+01 | 3.57E-01 |
| d13C_30 | -2.61E+01 | 5.63E-01 | -2.65E+01 | 9.78E-01 | -2.63E+01 | 1.56E+00 | -2.67E+01 | 6.36E-01 |  |  |  |  |  | 7.57E-01 | -2.67E+01 | 2.43E-01 |
| N_5 | 1.59E-01 | 3.83E-02 | 1.51E-01 | 4.16E-02 | 1.35E-01 | 1.60E-02 | 1.14E-01 | 1.85E-02 |  |  |  |  |  | 2.03E-02 | 1.44E-01 | 2.07E-02 |
| N_10 | 1.63E-01 | 5.28E-02 | 1.29E-01 | 2.42E-02 | 1.25E-01 | 1.77E-02 | 1.03E-01 | 1.83E-02 |  |  |  |  |  | 1.77E-02 | 1.38E-01 | 3.77E-02 |
| N_20 | 1.60E-01 | 4.50E-02 | 1.29E-01 | 2.47E-02 | 1.30E-01 | 1.85E-02 | 1.01E-01 | 2.03E-02 |  |  |  |  |  | 1.77E-02 | 1.25E-01 | 1.60E-02 |
| N_30 | 1.44E-01 | 5.55E-02 | 1.20E-01 | 3.93E-02 | 1.28E-01 | 2.31E-02 | 1.06E-01 | 3.02E-02 |  |  |  |  |  | 3.58E-02 | 1.10E-01 | 2.93E-02 |
| d15N_5 | 7.22E+00 | 6.71E-01 | 6.86E+00 | 8.06E-01 | 6.86E+00 | 6.66E-01 | 6.72E+00 | 1.04E+00 |  |  |  |  |  | 5.27E-01 | 6.14E+00 | 4.93E-01 |
| d15N_10 | 4.97E+00 | 3.10E-01 | 5.15E+00 | 6.06E-01 | 4.76E+00 | 6.21E-01 | 4.26E+00 | 7.37E-01 |  |  |  |  |  | 3.24E-01 | 3.96E+00 | 4.23E-01 |
| d15N_20 | 8.89E+00 | 3.09E-01 | 4.55E+00 | 2.64E+00 | 3.00E+00 | 3.01E-01 | 3.18E+00 | 4.55E-01 |  |  |  |  |  | 5.37E-01 | 3.93E+00 | 3.49E-01 |
| d15N_30 | 5.24E+00 | 1.64E-01 | 5.59E+00 | 5.07E-01 | 6.07E+00 | 4.85E-01 | 5.61E+00 | 1.12E+00 |  |  |  |  |  | 6.73E-01 | 4.91E+00 | 5.36E-01 |
| C/N_5 | 1.20E+01 | 3.11E+00 | 1.09E+01 | 4.86E-01 | 1.10E+01 | 2.05E-01 | 1.18E+01 | 1.49E+00 |  |  |  |  |  | 4.27E-01 | 1.05E+01 | 5.05E-01 |
| C/N_10 | 1.14E+01 | 1.08E+00 | 1.10E+01 | 4.63E-01 | 1.08E+01 | 4.12E-01 | 1.12E+01 | 3.55E-01 |  |  |  |  |  | 3.19E-01 | 1.10E+01 | 3.71E-01 |
| C/N_20 | 1.12E+01 | 1.22E+00 | 1.09E+01 | 9.02E-01 | 1.04E+01 | 2.07E-01 | 1.06E+01 | 2.66E-01 |  |  |  |  |  | 2.36E-01 | 1.04E+01 | 3.16E-01 |
| C/N_30 | 1.11E+01 | 1.28E+00 | 9.82E+00 | 6.90E-01 | 1.01E+01 | 3.84E-01 | 1.01E+01 | 1.91E-01 |  |  |  |  |  | 2.34E+00 | 1.07E+01 | 4.30E-01 |
| pH_5 | 7.18E+00 | 1.12E-01 | 7.14E+00 | 1.41E-01 | 6.99E+00 | 6.29E-02 | 6.80E+00 | 3.26E-01 |  |  |  |  |  | 1.54E-01 | 6.94E+00 | 1.35E-01 |
| pH_10 | 7.21E+00 | 1.18E-01 | 7.22E+00 | 1.07E-01 | 7.00E+00 | 9.87E-02 | 6.82E+00 | 4.39E-01 |  |  |  |  |  | 1.21E-01 | 6.95E+00 | 1.19E-01 |
| pH_20 | 7.26E+00 | 9.47E-02 | 7.23E+00 | 1.06E-01 | 7.04E+00 | 1.07E-01 | 6.68E+00 | 6.64E-01 |  |  |  |  |  | 1.23E-01 | 6.99E+00 | 9.88E-02 |
| pH_30 | 7.23E+00 | 1.46E-01 | 7.18E+00 | 1.09E-01 | 7.05E+00 | 1.14E-01 | 6.77E+00 | 4.80E-01 |  |  |  |  |  | 1.14E-01 | 6.98E+00 | 1.19E-01 |

| Plot | 5 | sd | 6 | sd | 7 | sd | 8 | sd |
| --- | --- | --- | --- | --- | --- | --- | --- | --- |
| K_S_ | 5.17E-03 | 7.96E-04 | 4.20E-03 | 1.04E-03 | 2.63E-03 | 1.12E-03 | 3.18E-03 | 1.19E-03 |
| IR | 5.06E-01 | 1.14E-01 | 4.06E-01 | 9.90E-02 | 4.50E-01 | 1.33E-01 | 5.13E-01 | 1.43E-01 |
| DB_15 | 1.39E+00 | 6.83E-02 | 1.41E+00 | 2.75E-02 | 1.41E+00 | 1.13E-01 | 1.39E+00 | 7.97E-02 |
| DB_30 | 1.52E+00 | 5.90E-02 | 1.56E+00 | 8.40E-02 | 1.53E+00 | 5.39E-02 | 1.48E+00 | 9.12E-02 |
| WHC_15 | 2.69E-01 | 1.54E-02 | 2.89E-01 | 2.71E-02 | 2.97E-01 | 1.94E-02 | 3.17E-01 | 2.59E-02 |
| WHC_30 | 2.44E-01 | 3.00E-02 | 2.46E-01 | 2.66E-02 | 2.64E-01 | 5.46E-02 | 3.16E-01 | 2.32E-02 |
| SOC_5 | 1.26E+00 | 2.34E-01 | 1.31E+00 | 1.78E-01 | 1.38E+00 | 2.17E-01 | 1.51E+00 | 2.35E-01 |
| SOC_10 | 1.00E+00 | 1.05E-01 | 1.13E+00 | 1.53E-01 | 1.31E+00 | 1.94E-01 | 1.55E+00 | 4.73E-01 |
| SOC_20 | 9.60E-01 | 1.62E-01 | 1.10E+00 | 2.17E-01 | 1.22E+00 | 1.83E-01 | 1.29E+00 | 1.81E-01 |
| SOC_30 | 7.25E-01 | 1.30E-01 | 1.10E+00 | 2.08E-01 | 1.07E+00 | 3.44E-01 | 1.19E+00 | 3.40E-01 |
| CO2_15 | 8.04E-02 | 1.18E-02 | 1.03E-01 | 2.57E-02 | 1.00E-01 | 1.41E-02 | 9.97E-02 | 2.23E-02 |
| CO2_30 | 5.56E-02 | 1.14E-02 | 6.77E-02 | 2.67E-02 | 7.54E-02 | 2.07E-02 | 8.21E-02 | 2.93E-02 |
| d13C_5 | -2.71E+01 | 4.37E-01 | -2.73E+01 | 4.98E-01 | -2.69E+01 | 2.55E-01 | -2.72E+01 | 3.40E-01 |
| d13C_10 | -2.68E+01 | 2.88E-01 | -2.69E+01 | 3.57E-01 | -2.69E+01 | 4.54E-01 | -2.68E+01 | 3.61E-01 |
| d13C_20 | -2.68E+01 | 3.54E-01 | -2.68E+01 | 5.30E-01 | -2.68E+01 | 6.59E-01 | -2.67E+01 | 3.57E-01 |
| d13C_30 | -2.65E+01 | 1.49E-01 | -2.70E+01 | 2.25E-01 | -2.65E+01 | 7.57E-01 | -2.67E+01 | 2.43E-01 |
| N_5 | 1.13E-01 | 1.75E-02 | 1.19E-01 | 1.36E-02 | 1.29E-01 | 2.03E-02 | 1.44E-01 | 2.07E-02 |
| N_10 | 8.63E-02 | 1.19E-02 | 9.75E-02 | 1.49E-02 | 1.24E-01 | 1.77E-02 | 1.38E-01 | 3.77E-02 |
| N_20 | 8.75E-02 | 1.28E-02 | 1.04E-01 | 1.69E-02 | 1.15E-01 | 1.77E-02 | 1.25E-01 | 1.60E-02 |
| N_30 | 7.50E-02 | 1.20E-02 | 1.03E-01 | 1.91E-02 | 9.63E-02 | 3.58E-02 | 1.10E-01 | 2.93E-02 |
| d15N_5 | 6.90E+00 | 1.13E+00 | 6.11E+00 | 5.45E-01 | 6.41E+00 | 5.27E-01 | 6.14E+00 | 4.93E-01 |
| d15N_10 | 4.52E+00 | 5.82E-01 | 3.79E+00 | 4.78E-01 | 4.19E+00 | 3.24E-01 | 3.96E+00 | 4.23E-01 |
| d15N_20 | 3.62E+00 | 8.76E-01 | 4.30E+00 | 5.92E-01 | 4.33E+00 | 5.37E-01 | 3.93E+00 | 3.49E-01 |
| d15N_30 | 5.18E+00 | 8.75E-01 | 5.34E+00 | 6.12E-01 | 5.07E+00 | 6.73E-01 | 4.91E+00 | 5.36E-01 |
| C/N_5 | 1.14E+01 | 9.17E-01 | 1.09E+01 | 4.33E-01 | 1.08E+01 | 4.27E-01 | 1.05E+01 | 5.05E-01 |
| C/N_10 | 1.19E+01 | 4.21E-01 | 1.16E+01 | 2.62E-01 | 1.06E+01 | 3.19E-01 | 1.10E+01 | 3.71E-01 |
| C/N_20 | 1.11E+01 | 3.49E-01 | 1.04E+01 | 6.18E-01 | 1.04E+01 | 2.36E-01 | 1.04E+01 | 3.16E-01 |
| C/N_30 | 9.82E+00 | 2.41E-01 | 1.07E+01 | 5.33E-01 | 1.14E+01 | 2.34E+00 | 1.07E+01 | 4.30E-01 |
| pH_5 | 6.43E+00 | 1.41E-01 | 7.08E+00 | 1.34E-01 | 6.91E+00 | 1.54E-01 | 6.94E+00 | 1.35E-01 |
| pH_10 | 6.24E+00 | 3.68E-01 | 7.10E+00 | 1.93E-01 | 6.97E+00 | 1.21E-01 | 6.95E+00 | 1.19E-01 |
| pH_20 | 6.44E+00 | 2.39E-01 | 7.23E+00 | 1.83E-01 | 6.99E+00 | 1.23E-01 | 6.99E+00 | 9.88E-02 |
| pH_30 | 6.43E+00 | 2.62E-01 | 7.18E+00 | 1.13E-01 | 7.00E+00 | 1.14E-01 | 6.98E+00 | 1.19E-01 |
